# An Autoantigen-ome from HS-Sultan B-Lymphoblasts Offers a Molecular Map for Investigating Autoimmune Sequelae of COVID-19

**DOI:** 10.1101/2021.04.05.438500

**Published:** 2021-04-06

**Authors:** Julia Y. Wang, Wei Zhang, Victor B. Roehrl, Michael W. Roehrl, Michael H. Roehrl

**Affiliations:** 1 Curandis, New York, USA; 2 Department of Gastroenterology, Affiliated Hospital of Guizhou Medical University, Guizhou, China; 3 Department of Pathology, Memorial Sloan Kettering Cancer Center, New York, USA; 4 Human Oncology and Pathogenesis Program, Memorial Sloan Kettering Cancer Center, New York, USA

**Keywords:** Autoantigens, autoantibodies, autoimmunity, SARS-CoV-2, COVID, Epstein-Barr virus

## Abstract

To understand how COVID-19 may induce autoimmune diseases, we have been compiling an atlas of COVID-autoantigens (autoAgs). Using dermatan sulfate (DS) affinity enrichment of autoantigenic proteins extracted from HS-Sultan lymphoblasts, we identified 362 DS-affinity proteins, of which at least 201 (56%) are confirmed autoAgs. Comparison with available multi-omic COVID data shows that 315 (87%) of the 362 proteins are affected in SARS-CoV-2 infection via altered expression, interaction with viral components, or modification by phosphorylation or ubiquitination, at least 186 (59%) of which are known autoAgs. These proteins are associated with gene expression, mRNA processing, mRNA splicing, translation, protein folding, vesicles, and chromosome organization. Numerous nuclear autoAgs were identified, including both classical ANAs and ENAs of systemic autoimmune diseases and unique autoAgs involved in the DNA replication fork, mitotic cell cycle, or telomerase maintenance. We also identified many uncommon autoAgs involved in nucleic acid and peptide biosynthesis and nucleocytoplasmic transport, such as aminoacyl-tRNA synthetases. In addition, this study found autoAgs that potentially interact with multiple SARS-CoV-2 Nsp and Orf components, including CCT/TriC chaperonin, insulin degrading enzyme, platelet-activating factor acetylhydrolase, and the ezrin-moesin-radixin family. Furthermore, B-cell-specific IgM-associated ER complex (including MBZ1, BiP, heat shock proteins, and protein disulfide-isomerases) is enriched by DS-affinity and up-regulated in B-cells of COVID-19 patients, and a similar IgH-associated ER complex was also identified in autoreactive pre-B1 cells in our previous study, which suggests a role of autoreactive B1 cells in COVID-19 that merits further investigation. In summary, this study demonstrates that virally infected cells are characterized by alterations of proteins with propensity to become autoAgs, thereby providing a possible explanation for infection-induced autoimmunity. The COVID autoantigen-ome provides a valuable molecular resource and map for investigation of COVID-related autoimmune sequelae and considerations for vaccine design.

## Introduction

The novel coronavirus SARS-CoV-2 has caused the worldwide COVID-19 pandemic with hundreds of millions infected and high morbidity and mortality. A significant proportion of patients who have recovered from the acute viral infection of COVID-19 continue to suffer from lingering health problems, so called “long COVID” syndrome. It is yet unknown how long the COVID aftereffects will persist, and more importantly, what the underlying causative mechanisms of long COVID syndrome are. The acute phase of COVID-19 is accompanied by various autoimmune responses, and autoimmune diseases, which tend to be chronic and debilitating, are major concerns of COVID-19 sequelae. To understand how SARS-CoV-2 infection may induce autoimmunity and how diverse the autoimmune disorders could be, we have started to compile a comprehensive atlas of COVID autoantigens (autoAgs) and autoantibodies (autoAbs), the root elements of autoimmunity [1, 2].

We have developed a unique DS-affinity enrichment strategy for autoAg discovery [1–8]. We discovered that dermatan sulfate (DS), a glycosaminoglycan that is abundant in skin and soft connective tissues and that is involved in wound healing and tissue repair, has affinity to autoAgs [3, 4]. Because of this affinity, DS binds autoAgs to form non-covalent DS-autoAg complexes, which transforms non-antigenic singular self-molecules into antigenic non-self-complexes [3]. DS-autoAg complexes are capable of engaging autoreactive B-cell receptors (autoBCRs) via a two-step process: (i) DS-autoAg complexes bind autoBCRs on autoreactive B1-cells via autoAg-autoBCR specificity; (ii) DS enters cells by (DS-autoAg)-autoBCR complex internalization and recruits a cascade of molecules to stimulate autoreactive B1-cells [3, 5]. In particular, DS recruits GTF2I that is required for IGH gene expression and IgH-associated multiprotein complexes in the endoplasmic reticulum (ER) to facilitate autoAb production [5]. Therefore, any self-molecule with DS-affinity has a propensity to be transformed by DS into an autoantigenic DS-autoAg complex [5]. Based on this unifying principle of DS-autoAg affinity, we have discovered and catalogued known and putative autoAgs from different cells and organs [1, 2, 6–8].

Autoantibodies, which target autoAgs, have been found in a significant portion of COVID-19 patients. In a cohort study of 147 hospitalized COVID-19 patients, autoAbs are detected in ~50% of the patients, and antinuclear autoAbs are detected in ~25% of patients, with the target autoAgs associated with myositis, systemic sclerosis, and connective tissue disease overlap syndromes [9]. In another study of 987 COVID-19 patients with life-threatening pneumonia, over 10% developed autoAbs against interferons, which likely neutralized their ability to block SARS-CoV-2 infection [10]. Although COVID-19 is typically mild or asymptomatic in children, multisystem inflammatory syndrome and multiple autoAbs developed in a portion of infected children [11, 12]. In a study of COVID-19 patients with unexplained neurological symptoms, anti-neuronal autoAbs were detected in sera or cerebrospinal fluid of all patients [13]. Antinuclear autoAbs, the most frequently tested autoAbs in clinical screening for autoimmune diseases such as lupus, Sjögren syndrome, scleroderma, or polymyositis, are found in 20–50% of COVID-19 patients [14–16].

Viral infections have long been regarded as culprits of autoimmune diseases. However, it has remained unclear how infections induce autoimmune diseases. In this study, we investigated HS-Sultan cells, a B-cell lymphoblast line isolated from Burkitt’s lymphoma of a 7-year-old boy. HS-Sultan cells are infected with and immortalized by Epstein-Barr virus (EBV) and carry the viral DNA sequence. From the proteome extracts of HS-Sultan cells, we identified a putative DS-affinity autoantigen-ome of 362 proteins, of which 201 are confirmed autoAgs by means of corresponding specific autoAbs reported in the literature. By comparing this autoantigen-ome with proteins affected by SARS-CoV-2 infection derived from multi-omic studies compiled in Coronascape [17–38], we identified 315 DS-affinity proteins and 186 confirmed autoAgs. When host cells are infected, numerous molecules undergo significant changes via altered expression, modification, or degradation. When the infected cells die, these altered molecules are released, and those with DS-affinity may become associated with DS and transform into immunogenic autoAg-DS complexes. This study illustrates that viral infections can profoundly change the host cell autoantigen-ome, result in a large repertoire of potential autoAgs, and may consequently lead to autoimmune disease.

## Results and Discussion

### DS-affinity autoantigen-ome of HS-Sultan cells

HS-Sultan cells were cultured, harvested, and lysed. Total proteins were extracted from lysates and fractionated on DS-Sepharose affinity resins. Proteins with no or weak DS-affinity were removed from the resins with 0.2 M NaCl, and those with intermediate to strong DS-affinity were eluted first with 0.5 M and then with 1.0 M NaCl. Proteins in the DS-affinity fractions were collected, desalted, digested, and sequenced by mass spectrometry. A total of 362 proteins were identified, with the majority present in the 0.5 M NaCl elution. Proteins that eluted with 1.0 M NaCl possess very strong DS-affinity and include some of the classical nuclear autoAgs, e.g., histones, TOP1, Sm-D3, and 60S acidic ribosomal protein P0. Other proteins with strong DS-affinity include both known autoAgs (vimentin, ATP synthetase ATP5B, and PABPC1) and unknown ones (RPL10A, L15, RPS27A, and mitochondrial single-stranded DNA binding protein SSBP1).

Of the 362 DS-affinity proteins identified from HS-Sultan cells, 201 (55.5%) are confirmed humoral autoAgs based on prior literature reports of specific autoantibodies (see references in Table 1). These autoAgs and their respective autoantibodies are found in a wide spectrum of autoimmune diseases and cancer. The number of actual autoAgs is likely much greater, as most of the unconfirmed proteins have structural resemblance to known autoAgs. For example, SSBP1 is structurally and functionally similar to the classical lupus autoAg SSB, but it has not formally been identified as an autoAg. As another example, nucleosome assembly protein 1-like 1 and 4 (NAP1L1, NAP1L4) are identified by DS-affinity but unconfirmed as autoAgs, whereas their close relative NAP1L3 has been reported as an autoAg. Due to the structural similarity of many DS-affinity proteins with known autoAgs, it is likely that there are additional yet-to-be discovered (considered putative) autoAgs in this group.

**Fig. 1.**
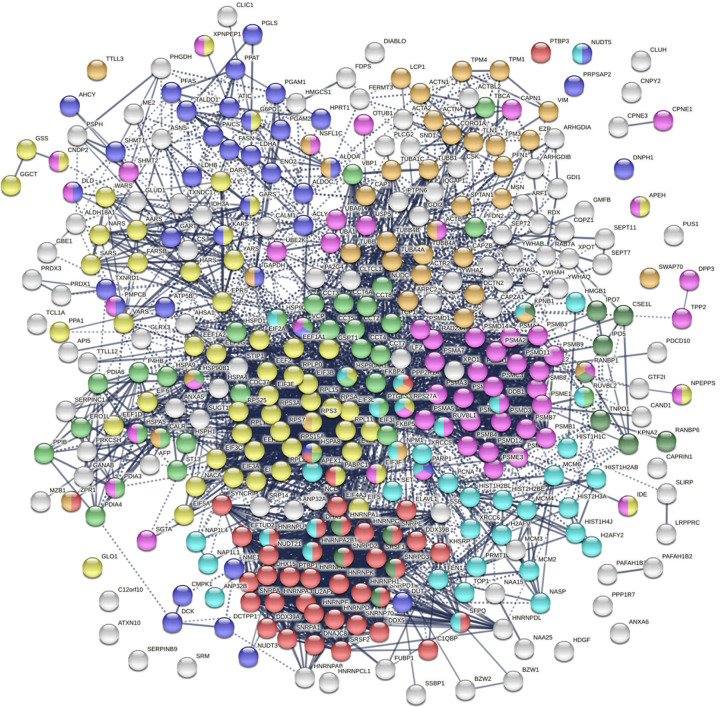
The autoantigen-ome of HS-Sultan cells identified by DS-affinity. Marked proteins are associated with peptide biosynthesis and catabolic process (58 proteins, yellow), protein folding (34 proteins, light green), RNA splicing (41 proteins, red), nucleobase-containing small molecule metabolic process (39 proteins, blue), proteolysis (55 proteins, pink), import into nuclear (13 proteins, dark green), cytoskeleton organization (39 proteins, amber), and chromosome organization (40 proteins, aqua).

**Fig. 2.**
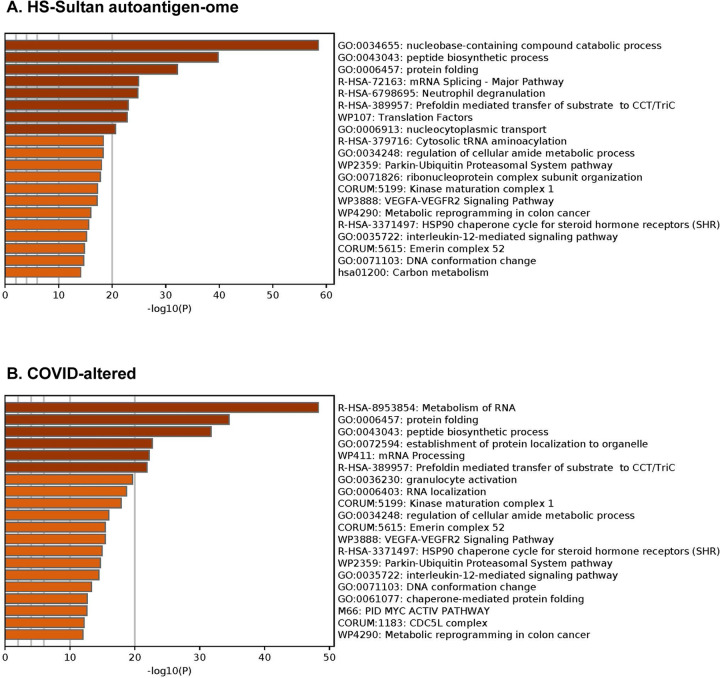
Top 20 enriched pathways and processes among COVID-altered autoAgs. Top: Pathways of 362 proteins identified by DS-affinity from HS-Sultan cells. Bottom: Pathways of 316 DS-affinity proteins that are altered in COVID.

The DS-affinity autoantigen-ome, which includes confirmed and putative autoAgs, is not a random collection of proteins but functionally highly connected. As shown by protein-protein interaction analysis, the DS-affinity autoantigen-ome possesses significantly more interactions than expected (3,105 interactions at high confidence level vs. 1,249 expected; PPI enrichment p-value <1.0E-16). Based on Gene Ontology (GO) biological process analysis, the DS-affinity autoantigen-ome of HS-Sultan cells is significantly associated with RNA slicing, translation, peptide biosynthesis, protein folding, proteolysis, biosynthesis and metabolism of nucleobase-containing small molecules (e.g., nucleobase, nucleoside, and nucleotide phosphate), cytoskeleton organization, and chromosome organization (Fig. 1). Pathway and process enrichment analysis reveals that it is also significantly associated with neutrophil degranulation, nucleocytoplasmic transport, kinase maturation complex, metabolic reprogramming, and IL-12 mediated signaling (Fig. 2A).

The DS-affinity autoantigen-ome is dominated by several families of proteins. There are 24 proteasomal proteins, 22 spliceosome proteins, 14 hnRNPs, 13 aminoacyl-tRNA synthases (ligases), 13 translation initiation factors subunits, 12 ribosomal proteins, 10 heat shock proteins, 9 actin and actin-related proteins, 9 tubulins, 8 histones, 8 snRNPs, 7 T-complex proteins (CCT/TriC), 6 elongation factor subunits, and 6 14-3-3 proteins. The majority of the proteins in these families have been reported as autoAgs (Table 1). For example, all hnRNP and snRNP proteins identified by DS-affinity in this study are among the best-known nuclear autoAgs. Interestingly, autoAgs included in clinical diagnostic autoimmune disease ANA screening panels, such as SSB (lupus La), SNRPD1 (Sm D1), SNRPD3 (Sm D3), histones, and TOP1, are all identified in this study by DS-affinity enrichment from HS-Sultan cells.

In addition to proteins, such as ribosomal and ribonucleoproteins, that can be consistently identified from a variety of cell types, HS-Sultan B lymphoblast cells give rise to a large number of unique DS-affinity protein categories. In particular, many proteins associated with biomolecule biosynthesis are identified. Among them are proteins involved in inosine monophosphate and purine nucleotide biosynthesis (ATIC, GART, HPRT1, PAICS, PFAS, PPAT, SHMT1), amino acid biosynthesis (CS, IDH3A, PHGDH, PGAM1, PGAM2, PSPH), and carbohydrate biosynthesis and catabolic processes (ALDOA, ALDOC, ENO2, G6PD, GBE1, LDHA, TALDO1). There are also proteins involved in protein transport (ARF1, CSE1L, GDI1, GDI2, HMGB1, IPO5, KPNA2, RAB7A, RANBP1, RANBP6, SRP14, TNPO1, XPOT), dephosphorylation (NSF1C, PPP1R7, PPP2R1A, SET, SWAP70), and ubiquitination and de-ubiquitination (OTUB1, SHMT2, UBA1, UBA6, UBE2K, USP5). 17 of these 44 proteins are currently known autoAgs, while the remainder await further investigation. Overall, HS-Sultan cells appear to be especially rich in biosynthetic protein machinery, which may explain the rapid proliferation of these cells in Burkitt lymphoma.

Thirteen aminoacyl-tRNA synthetases were identified by DS-affinity from HS-Sultan cells, including AARS, DARS, ERPS, FARSB, GARS, HARS, KARS, NARS, PUS1, SARS, VARS, WARS, and YARS. Ten of these are already known autoAgs (Table 1), although we suspect that the remainder will also likely be autoAgs. This group of proteins are the culprits of antisynthetase syndrome, an autoimmune disease characterized by autoantibodies against one or multiple tRNA synthetases. Antisynthetase syndrome is a chronic disorder that can affect many parts of the body, with common symptoms including myositis, interstitial lung disease, polyarthritis, skin thickening and cracking of fingers and toes, or Raynaud disease. Antisynthetase syndrome has been reported in a case report of COVID-19 interstitial lung disease [39].

HS-Sultan cells are B lymphoblasts immortalized by Epstein-Barr virus (EBV) infection and carry viral DNA sequences. Using DS-affinity, we identified numerous proteins involved in DNA repair and the mitotic cell cycle, including CLTC, DCTN2, MCM2, MCM3, MCM4, MCM6, NSF1C, PNCA, PPAT, and SUGT1. Using DS-affinity, we also identified many proteins associated with telomerase maintenance, including TCP1, CCT2, CCT4, CCT5, CCT7, HNRNPA1, HNRPNA2B1, HNRNPC, HNRNPD, HNRNPU, HSP90AA1, HSP90AB1, PARP1, PTGES3, and XRCC5. Telomerase maintenance, which counteracts DNA damage response, cell cycle arrest, and apoptosis, is crucial for immortalization of cells with unlimited proliferative potential. Of these 25 proteins, 19 are known autoAgs, which indicates that proteins involved in telomerase maintenance, DNA repair, and cell cycle may be affected by EBV infection and become autoantigenic.

### DS-affinity autoantigen-ome related to SARS-CoV-2 infection

To find out whether DS-affinity autoAgs are affected in SARS-CoV-2 infection, we conducted similarity searches with currently available multi-omic COVID-19 data compiled in Coronascape (as of 02/22/2021) [17–38]. Among our 362 DS-affinity proteins, 315 (87.0%) are affected by SARS-CoV-2 infection (Table 1). Of these 315 proteins, 209 are up-regulated and 248 are down-regulated at protein and/or mRNA levels, and 95 are in the interactomes of individual SARS-CoV-2 viral proteins. Because the COVID-19 multi-omics data have been obtained with various methods from different infected cells or patients, there are proteins found up-regulated in some studies but down-regulated in other studies, but nevertheless, these proteins are affected by the infection and thus considered COVID-affected in our analysis (Supplemental Table 1). Of the 315 COVID-affected DS-affinity proteins, 186 (59.0%) are thus far confirmed autoAgs, while 129 are putative autoAgs (Table 1).

**Fig. 3.**
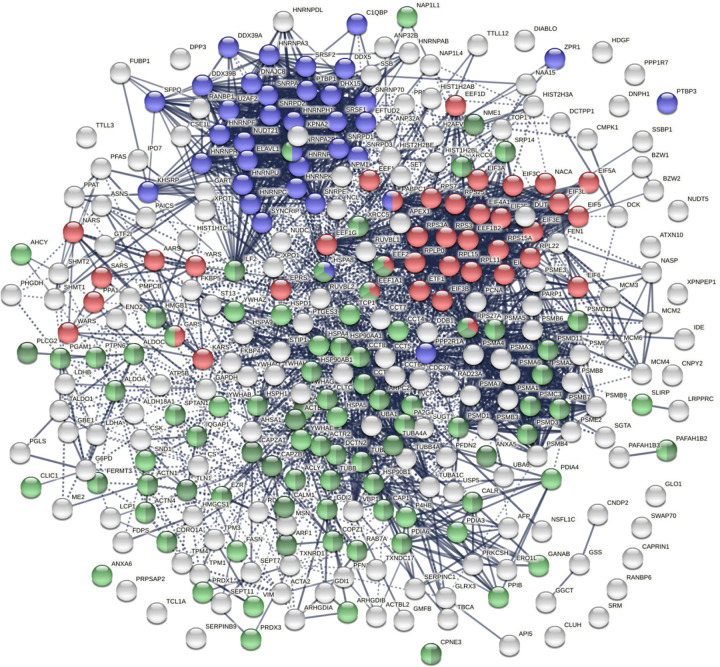
COVID-affected proteins shared with the HS-Sultan autoantigen-ome. Marked proteins are associated with RNA splicing (36 proteins, blue), translation (39 proteins, red), vesicle (77 proteins, green) and vesicle-mediated transport (62 proteins, dark green).

The COVID-affected DS-affinity proteins are highly connected (Fig. 3). By STRING analysis, these 315 proteins exhibit 2,507 interactions at high confidence level, which is significantly higher than randomly expected (1,002 interactions) with PPI enrichment p-value <1.0E-16. The proteins are primarily associated with RNA and mRNA processing, translation, vesicles, and vesicle-mediated transport (Fig. 3), which is consistent with our findings in other cell types [1, 2, 8]. In addition, these proteins are enriched in protein folding, peptide biosynthesis, granulocyte activation, emerin complex, IL-12 mediated signaling pathway, CDC5L complex, and metabolic reprogramming (Fig. 2B).

Twenty-one COVID-affected DS-affinity proteins are associated with mRNA splicing, including heterogenous nuclear ribonucleoproteins (hnRNP A1, A2B1, A3, AB, C, DL, F, H1, K, Q, R, and U), small nuclear ribonucleoproteins (SNRNP70, SNRPA, SNRPE, SNRPD1, SNRRPD2, SNRPD3), and splicing factors (SRSF1, SRSF2, SFPQ), all of which are well-known autoAgs.

**Fig. 4.**
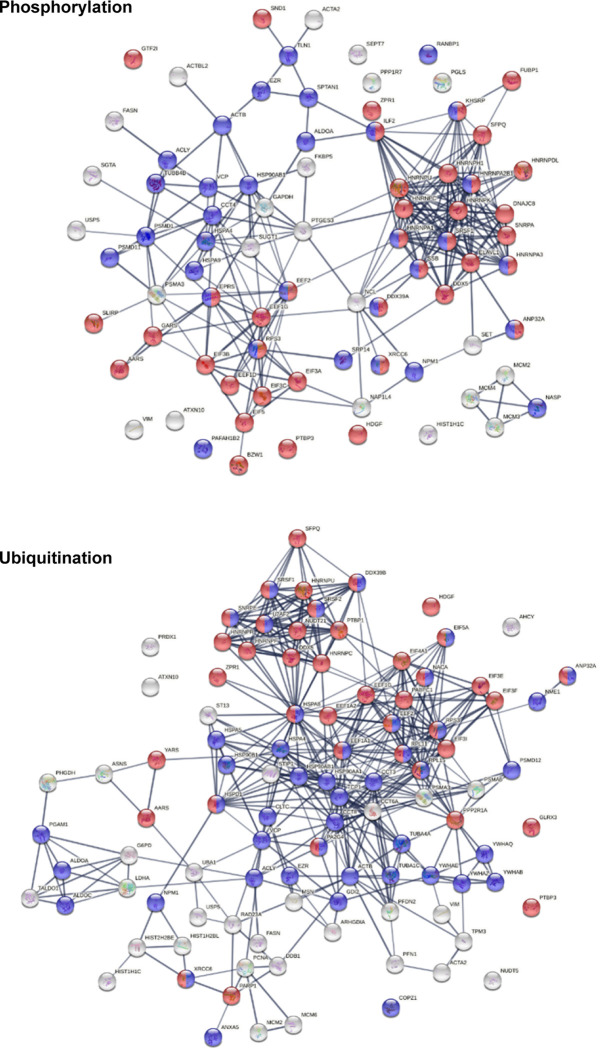
DS-affinity proteins that are altered by phosphorylation or ubiquitination in SARS-CoV-2 infection. Marked proteins are associated with gene expression (red) and transport (blue).

Phosphorylation and ubiquitination changes induced by SARS-CoV-2 infection are posttranslational molecular alterations that may transform native proteins into potential autoAgs (Fig. 4), which is consistent with our previous findings [2]. Phosphorylation changes affected 80 COVID-altered DS-affinity proteins, including 8 hnRNPs, 4 initiation factors (EIF3A, 3B, 5), 3 elongation factors, 3 replication licensing factors (MCM2, 3, 4), SSB, XRCC6, and GTF2I. These phosphoproteins are associated with mRNA splicing, translation, telomere maintenance, DNA conformation change, and pre-replicative complex assembly. Ubiquitination changes affected 101 COVID-altered DS-affinity proteins, including 8 heat shock proteins, 5 initiation factors (EIF3E, 3F, 3I, 4A1, 5A), 4 CCT units, 4 14-3-3 proteins, 3 elongation factors, 3 histones, and 2 MCMS. These ubiquitinated proteins are associated with the nucleobase-containing compound catabolic process, RNA metabolism, cellular response to stress, prefoldin mediated transfer of substrate to CCT/TriC, and axon guidance.

### AutoAgs that interact with SARS-CoV-2 components

**Fig. 5.**
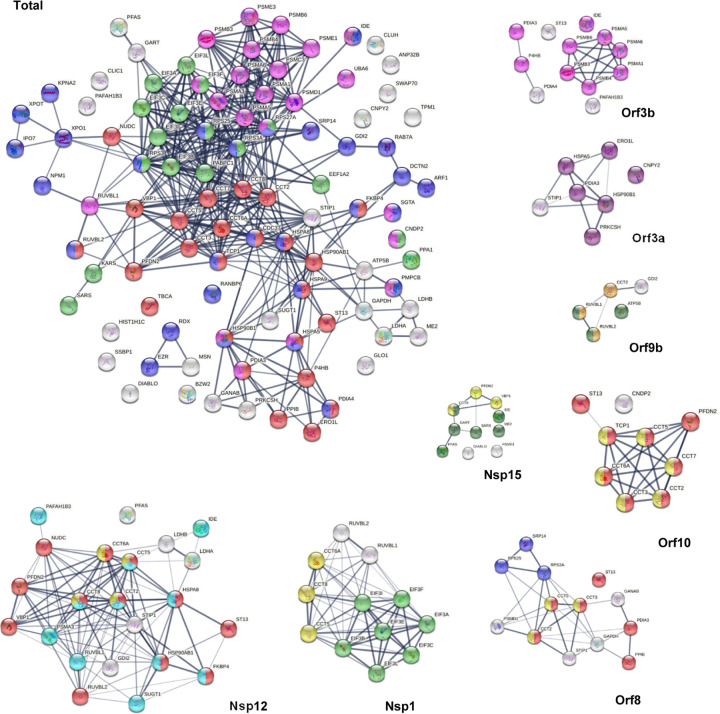
DS-affinity proteins in the SARS-CoV-2 interactomes. **Total**: marked proteins are involved in protein folding (25 proteins, red), peptide biosynthetic process (17 proteins, green), protein localization (29 proteins, blue), or proteolysis (22 proteins, pink). **Orf3b**: proteolysis (pink). **Orf3a**: endoplasmic reticulum (dark purple). **Orf9b**: nuclear function of prefoldin (amber), AAA+ ATPase domain or P-loop containing nucleoside triphosphate hydrolase (dark green). **Nsp15**: prefoldin-mediated transfer of substrate to CCT/TriC (yellow), nucleotide binding (dark green). **Orf10**: protein folding (red), CCT chaperonin (yellow). **Orf8**: protein folding (red), SRP-dependent cotranslational protein targeting to membrane (blue), CCT chaperonin (yellow). **Nsp1**: translation initiation (green), CCT chaperonin (yellow). **Nsp12**: protein folding (red), multi-organism process (aqua), CCT chaperonin (yellow).

There are 95 DS-affinity proteins found in the interactomes of various SARS-CoV-2 proteins (Fig. 5), meaning that these proteins can interact directly or indirectly with viral components. They appear to be intimately involved in protein metabolism, including 17 proteins related to peptide biosynthesis, 25 related to protein folding, 29 related to protein localization, and 22 related to proteolysis. In addition, these proteins are associated with the symbiont viral process, translational initiation, protein deubiquitination, protein stabilization, and posttranslational protein modification.

The chaperonin-containing T-complex (CCT), also known as T-complex protein ring complex (TriC), is the chaperonin of eukaryotic cells. The human CCT/TriC complex is a two-ring barrel-like complex formed by 8 similar but distinct subunits. Remarkably, all 8 CCT subunits are identified by DS-affinity, and 7 of them are found in the SARS-CoV-2 interactomes: CCT2 (interacts with Nsp12, Orf8, Orf9b, Orf10), CCT5 (with Nsp1, Nsp12, Orf8, Orf10), CCT6A (with Nsp1, Nsp12, Orf10), CCT8 (with Nsp1, Nsp12, Nsp14), CCT3 (with Orf8, Orf10), TCP1 (with Orf10), and CCT7 (with Orf10). In total, 6 SARS-CoV-2 proteins interact with the host cell CCT chaperonin, with Orf10 interacting with 6 CCT subunits (Fig. 5). Furthermore, CCT subunits and PDFN2 (prefoldin subunit 2) are found together in the interactomes of Orf10, Nsp12, and Nsp15. CCTs assist the folding of proteins upon ATP hydrolysis, aiding in the folding of ~10% of the proteome. PDFN2 binds the nascent polypeptide chain and promotes folding in an environment in which there are many competing pathways for nonnative proteins. Therefore, these findings suggest that SARS-CoV-2 exploits CCT complex and PDFN2 to ensure competitive folding of nonnative viral proteins in the host cells.

In addition to CCT/TriC, heat shock proteins (HSPs) are another group of chaperones frequently identified with DS-affinity. Ten HSPs are identified in this study, including HSPA4, HSPA5, HSPA8, HSPA9, HSPD1, HSPH1, HSP90AA1, HSP90AA2, HSP90AB1, and HSP9B1. All 10 are known autoAgs (Table 1). HSP8 interacts with Nsp2 and Nsp12. HSP90B1 (endoplasmin) interacts with Orf3a and Orf9c. HSPA9 interacts with N protein. HSP90AB1 interacts with Nsp12. Most strikingly, HSPA5 (GRP78, BiP) interacts with 9 SARS-CoV-2 proteins, including S, E, M, Nsp2, Nsp4, Nsp6, Orf3a, Orf7a, and Orf7b. In addition, CDC37 (Hsp90 co-chaperone, hsp90 chaperone protein kinase-targeting) interacts with Nsp16. ST13 (Hsc70-interacting protein) interacts with 5 SARS-CoV-2 proteins (Nsp12, Orf3b, Orf6, Orf8, and Orf10). STIP1 (stress induced phosphoprotein 1, HSP90AA1 co-chaperone) interacts with 4 viral proteins (Nsp12, Orf3a, Orf8, and E).

The replication machinery of SARS-CoV-2 interacts with 41 different DS-affinity proteins. Nsp12, an RNA-dependent RNA polymerase and the central component of the replication machinery, interacts with the largest number (i.e., 22) of DS-affinity proteins (Fig. 5). Its cofactor Nsp7 interacts with 12 proteins and Nsp8 interacts with only one. The replication machine also includes a helicase (Nsp13), 2 ribonucleases (Nsp14 and Nsp15), 2 RNA-cap methyltransferases (Nsp14, Nsp16), and cofactor Nsp10. Nsp15 interacts with 10 DS-affinity proteins, Nsp16 interacts with 8 proteins, Nsp13 interacts with SRP14 and RDX, Nsp14 interacts with IDE and CCT8, and Nsp10 interacts with PSMA3. Nsp12-interacting DS-affinity proteins are strongly associated with protein folding, particularly prefoldin mediated transfer of substrates to CCT complex and cooperation of prefoldin and CCT in protein folding (Fig. 5). Nsp15-interacting proteins are also associated with prefoldin-mediated substrate transfer to CCT. DS-affinity proteins interacting with other individual viral replication components have no clear biological associations.

Orf3b of SARS-CoV-2 interacts with 12 DS-affinity proteins, including 6 proteasomal proteins, 3 protein disulfide-isomerases, IDE, ST13, and PAFAH1B3 (Fig. 5). Orf3a interacts with 7 proteins, including STIP1 (stress-induced-phosphoprotein 1) and 6 ER proteins (HSPA5, HSP90B1, CNPY2, ERO1L, PRKCSH, and PDIA3). CANPY2 prevents MIR-mediated MRLC ubiquitination and its subsequent proteasomal degradation. ERO1L (or ERO1A) is an oxidoreductase in disulfide bond formation in the ER. PRKCSH (glucosidase II subunit beta) cleaves sequentially the 2 innermost glucose residues from the Glc_2_Man_9_GlcNAc_2_ oligosaccharide precursor of immature glycoproteins. Based on the normal functions of their interacting proteins, Orf3a and Orf3b appear to affect host stress response and protein processing in the ER.

The S protein of SARS-CoV-2 is found to interact with HSPA5 (GRP78/BiP), PRKCSH, PRS27A (ubiquitin-40S ribosomal protein), MSN, and EZR. EZR and MSN are members of the ezrin-moesin-radixin (ERM) family, and its third member RDX is found to interact with Nsp13 of the virus. Moesin is localized to filopodia and other membranous protrusions that are important for cell-cell recognition, and ERM proteins connect the plasma membranes to the actin-based cytoskeleton. Actin and cytoskeleton proteins have been consistently found to be altered in SARS-CoV-2 infection in our previous studies [1, 2], and this finding suggests that ERM proteins facilitate the viral trafficking from host cell membrane to the cytoskeleton. All three ERM proteins are confirmed autoAgs.

Nsp1 is a major virulence factor of coronavirus. COVID-19 patients with autoantibodies are found to have higher levels of antibodies against SARS-CoV-2 Nsp1 protein [9]. Nsp1 has been reported to hijack the host 40S ribosome by inserting its C terminus into the mRNA entry tunnel, which effectively blocks RGI-dependent innate immune responses [40]. In this study, we found that Nsp1 interacts with 7 subunits of the translation initiation factor 3 complex (EIF3 A, B, C, E, F, I, L). EIF3 complex binds the 40S ribosome and serves as a scaffold for other initiation factors, auxiliary factors, and mRNA. Hence, our study extends previous reported activities of Nsp1 and shows that Nsp1 engages both the 40S ribosome and EIF3 to manipulate host protein translation.

A few interesting SARS-CoV-2-interacting DS-affinity proteins may provide clues to potential COVID-19 symptoms. PAFAH1B3 is a catalytic unit of the platelet-activating factor acetylhydrolase complex and plays important roles in platelet activation regulation and brain development, and it interacts with Nsp12, Nsp5, and Orf3b. Another subunit, PAFAH1B2, is altered in SARS-CoV-2 infection. Both this and our previous studies [2] have identified PAFAH1B2 and B3 as potential COVID-altered autoAgs, and their roles in COVID coagulopathy merit further investigation.

IDE (insulin degrading enzyme) is a ubiquitously expressed metalloprotease that degrades insulin, beta amyloid, and others. IDE interacts with 6 SARS-CoV-2 proteins (Nsp4, Nsp12, Nsp14, Nsp15, Nsp16, and Orf3b). Although its role in COVID remains unknown, IDE has been partially characterized in other viral infections. It is one of the host factors of hepatitis C virus [41], and it degrades HIV-1 p6 Gap protein and regulates virus replication in an Env-dependent manner [42]. In varicella zoster virus infection, the viral gE protein precursor associates with IDE, HSPA5, HSPA8, HSPD1, and PPIA in the ER of infected cells [43]. Interestingly, this group of ER proteins is also identified in this study, although we identified PPIB instead of PPIA. Although IDE has not yet formally been described as an autoAg, we have identified IDE in this and another study [2], and its importance for COVID-19 and autoimmunity merits further investigation.

### DS-affinity and B-cell-specific IgH-ER complex

**Fig. 6.**
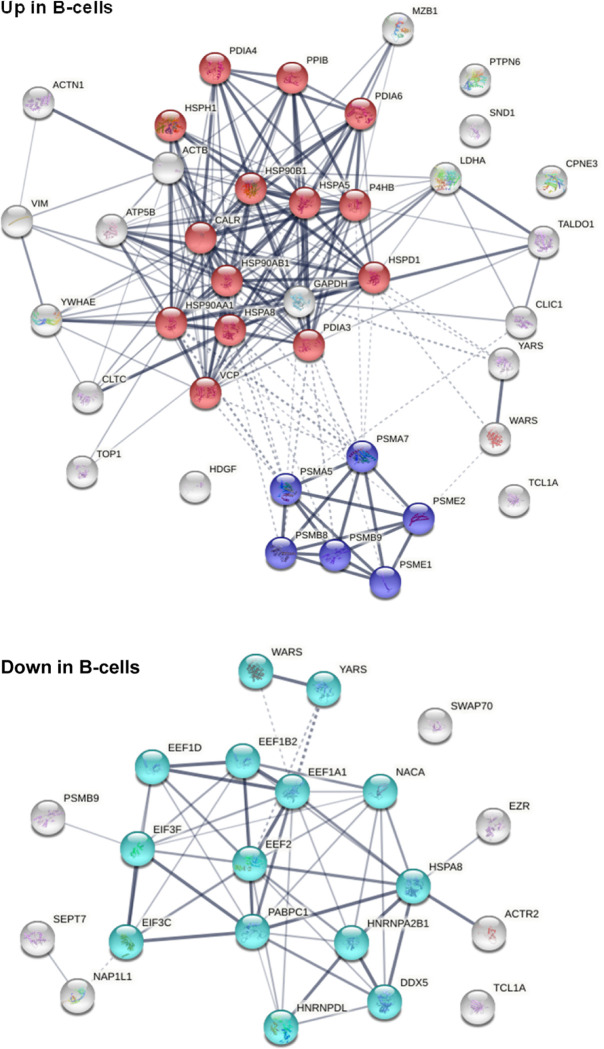
DS-affinity proteins from HS-Sultan cells that are up-regulated or down-regulated in B-cells of COVID-19 patients. **Top**: 39 up-regulated proteins. Red: protein processing in ER. Blue: proteasome. **Bottom**: 21 down-regulated proteins. Aqua: proteins involved in gene expression.

Because HS-Sultan cells are derived from B lymphoblasts infected by Epstein-Barr virus, we compared the DS-affinity autoantigen-ome with single-cell mRNA expression profiles of B-cells from 7 patients hospitalized with COVID-19 [23]. We identified 39 DS-affinity proteins that are up-regulated at mRNA level in COVID B-cells, which include 7 heat shock proteins, 6 proteasomal proteins, 4 protein disulfide-isomerases, HDGF (heparin binding growth factor), CLIC1, CPNE3, SND1, TALDO1, TCL1A, and others (Fig. 6). These up-regulated proteins are primarily associated with protein processing in the ER and the proteasome. We also identified 21 DS-affinity proteins that are down-regulated in COVID B-cells, including 4 translation elongation factors, 2 translation initiation factors, 2 hnRNPs, 2 aminoacyl-tRNA synthetases, NACA, NAP1L1, and PABPC1. These down-regulated proteins are primarily associated with gene expression (Fig. 6).

In particular, MZB1 (marginal zone B- and B1-cell-specific protein) is found up-regulated in B-cells from 5 of the 7 COVID-19 patients. MZB1 is a B-cell-specific ER-localized protein that is most abundantly expressed in marginal zone B-cells and B1-cells [44]. These cells are also termed innate-like B cells. They differ from follicular B-cells by their attenuated Ca^2+^ mobilization, fast antibody secretion, and increased cell adhesion. MZB1 plays important roles in humoral immunity and helps diversify peripheral B-cell functions by regulating calcium stores, antibody secretion, and integrin activation. MZB1 mRNA expression was found increased by >2-fold in B-cells of SLE patients with active disease [45]. High MZB1 gene expression predicted adverse prognosis in chronic lymphocytic leukemia, follicular lymphoma, and diffuse large B-cell lymphoma [46]. High prevalence of MZB1-positive plasma B-cells in tissue fibrosis was found in human lung and skin fibrosis, and MZB1 levels correlated positively with tissue IgG and negatively with diffusion capacity of the lung [47].

MZB1 is part of a B-cell-specific ER chaperone complex, associates with IgM heavy chain (IgH) and HSP90B1 (Grp94), and augments IgM assembly and secretion [48]. MZB1 is also found to augment the function of PDIA3 (ERp57) [44]. In this study, MZB1, HSP90B1, HSPA5 (Grp78, BiP), PDIA4, PDIA6, and CALR are found jointly up-regulated in the B-cells of the same 5 COVID-19 patients. This finding suggests that these 6 proteins are in the same IgH-associated ER complex. In addition, several other up-regulated ER proteins are identified, including HSP90AB1, HSPD1, HSPA8, PDIA3, P4HB, and PPIB.

Interestingly, in our study of murine pre-B1 lymphoblasts, we also found that DS interacts with the same IgH-associated multiprotein complex in the ER [5]. In addition, we had observed that DS interacts directly with GTF2I in murine pre-B1 cells, and GTF2I is also identified by DS-affinity in human B lymphoblast HS-Sultan cells in this study. GTF2I is a required gene transcription factor at the *IgH* gene locus. Pre-B1 cells, which express precursor B-cell receptors (preBCRs) that are polyreactive and autoreactive, are a critical check point in the development of mature autoreactive B cells. The Ig heavy chain (IgH) repertoire of autoantibodies is determined at the pre-B stage. Our previous findings from pre-B1 cells suggested that DS is a potential master regulator of IgH at both the gene and protein expression levels, i.e., DS recruits GTFI for *IgH* gene expression and engages IgH-associated ER complex for autoantibody production. The findings from this study provide further support for a key role of DS in regulating autoantibody production and autoreactive B1-cell development. Furthermore, the finding from B-cells of COVID-19 patients point to a potential significance of autoreactive B1 cells in COVID-induced autoimmunity.

## Conclusion

Exploiting the affinity between autoAgs and DS glycosaminoglycan, we identified 362 DS-affinity proteins from EBV-immortalized HS-Sultan cells. 201 of these DS-affinity proteins are already known autoAgs in a wide variety of autoimmune diseases and cancer. Of the 362, 315 DS-affinity proteins are affected by SARS-CoV-2 infection, and 186 COVID-affected DS-affinity proteins are known autoAgs. These COVID-altered proteins are largely affected by phosphorylation, ubiquitination, or interaction with viral protein components. They are associated with gene expression, mRNA processing, mRNA splicing, translation, protein folding, DNA replication fork, telomerase maintenance, chromosome organization, biosynthesis and catabolism of nucleobase-containing molecules and proteins, vesicles, and nucleocytoplasmic transport. CCT/TriC chaperonin, insulin degrading enzyme, and platelet-activating factor acetylhydrolase are found in the interactomes of multiple viral Nsp and Orf proteins. By integrating DS-affinity autoAgs with multi-omic data from COVID, our study suggests that viral infections can cause significant proteomic alterations, give rise to a diverse pool of autoAgs, and may lead to infection-induced autoimmune diseases. The COVID autoantigen-ome provided in this paper may serve as a molecular map and resource for investigating autoimmune phenomena of SARS-CoV-2 infection and its long-term sequelae. Understanding immunogenic proteins of COVID may also enhance vaccine target design.

## Materials and Methods

### HS-Sultan cell culture

The human B lymphoblast HS-Sultan cell line was obtained from the ATCC (Manassas, VA) and cultured in complete RPMI-1640 medium. The growth medium was supplemented with 10% fetal bovine serum and a penicillin-streptomycin-glutamine mixture (Thermo Fisher). The cells were grown at 37 °C in a CO_2_ incubator, and about 300 million cells were harvested for the study.

### Protein extraction

Protein extraction was performed as previously described [4]. In brief, HS-Sultan cells were lysed with 50 mM phosphate buffer (pH 7.4) containing the Roche Complete Mini protease inhibitor cocktail and then homogenized on ice with a microprobe sonicator until the turbid mixture turned nearly clear with no visible cells left. The homogenate was centrifuged at 10,000 g at 4 °C for 20 min, and the total protein extract in the supernatant was collected. Protein concentration was measured by absorbance at 280 nm using a NanoDrop UV-Vis spectrometer (Thermo Fisher).

### DS-Sepharose resin preparation

The DS-affinity resins were synthesized as previously described [4, 8]. In brief, 20 ml of EAH Sepharose 4B resins (GE Healthcare Life Sciences) were washed with distilled water three times and mixed with 100 mg of DS (Sigma-Aldrich) in 10 ml of 0.1 M MES buffer, pH 5.0. About 100 mg of N-(3-dimethylaminopropyl)-N’-ethylcarbodiimide hydrochloride (Sigma-Aldrich) powder was added, and another 100 mg was added after 8 h of reaction. The reaction proceeded by mixing on a rocker at 25 °C for 16 h. The coupled resins were washed with water and equilibrated with 0.5 M NaCl in 0.1 M acetate (pH 5.0) and 0.5 M NaCl in 0.1 M Tris (pH 8.0).

### DS-affinity fractionation

The total proteomes extracted from HS-Sultan cells were fractionated in a DS-Sepharose column in a manner similar to previously described [4]. About 40 mg of proteins in 40 ml of 10 mM phosphate buffer (pH 7.4; buffer A) were loaded onto the DS-affinity column at a rate of 1 ml/min. Unbound and weakly bound proteins were removed with 60 ml of buffer A and then 40 ml of 0.2 M NaCl in buffer A. The remaining bound proteins were eluted in steps with 40 ml 0.5 M NaCl and then with 40 ml 1.0 M NaCl in buffer A. Fractions were desalted and concentrated with 5-kDa cut-off Vivaspin centrifugal filters (Sartorius). Fractionated proteins were separated in 1-D SDS-PAGE in 4–12% Bis-Tris gels, and the gel lanes were divided into two or three sections for mass spectrometric sequencing.

### Mass spectrometry sequencing

Protein sequencing was performed at the Taplin Biological Mass Spectrometry Facility at Harvard Medical School. Proteins in gels were digested with sequencing-grade trypsin (Promega) at 4 °C for 45 min. Tryptic peptides were separated in a nano-scale C_18_ HPLC capillary column and analyzed in an LTQ linear ion-trap mass spectrometer (Thermo Fisher). Peptide sequences and protein identities were assigned by matching the measured fragmentation pattern with proteins or translated nucleotide databases using Sequest. All data were manually inspected. Proteins with ≥2 peptide matches were considered positively identified.

### COVID data comparison

DS-affinity proteins were compared with currently available COVID-19 multi-omic data compiled in the Coronascape database (as of 02/22/2021) [17–38]. These data have been obtained with proteomics, phosphoproteomics, interactome, ubiquitome, and RNA-seq techniques. Up- and down-regulated proteins or genes were identified by comparing cells infected vs. uninfected by SARS-CoV-2 or COVID-19 patients vs. healthy controls. Similarity searches were conducted to identify DS-affinity proteins that are up- and/or down-regulated in viral infection at any omic level.

### Protein network analysis

Protein-protein interactions were analyzed by STRING [49]. Interactions include both direct physical interaction and indirect functional associations, which are derived from genomic context predictions, high-throughput lab experiments, co-expression, automated text mining, and previous knowledge in databases. Each interaction is annotated with a confidence score from 0 to 1, with 1 being the highest, indicating the likelihood of an interaction to be true. Pathways and processes enrichment were analyzed with Metascape [17], which utilize various ontology sources such as KEGG Pathway, GO Biological Process, Reactome Gene Sets, Canonical Pathways, CORUM, TRRUST, and DiGenBase. All genes in the genome were used as the enrichment background. Terms with a p-value <0.01, a minimum count of 3, and an enrichment factor (ratio between the observed counts and the counts expected by chance) >1.5 were collected and grouped into clusters based on their membership similarities. The most statistically significant term within a cluster was chosen to represent the cluster.

### Autoantigen literature text mining

Every DS-affinity protein identified in this study was searched for specific autoantibodies reported in the PubMed literature. Search keywords included the MeSH keyword “autoantibodies”, the protein name or its gene symbol, or alternative names and symbols. Only proteins for which specific autoantibodies are reported in PubMed-listed journal articles were considered “confirmed” autoAgs in this study.

## Supplementary Material

Supplement 1

## Figures and Tables

**Table 1.** DS-affinity autoantigens from HS-Sultan cells and their alteration in COVID-19

Number of Peptides		Protein	Alteration in COVID (up and/or down)		SARS-CoV-2 interaction	DS-affinity (1.0 M)	DS-affinity (0.5 M)	Ref.
6	AARS	Alanine-tRNA ligase, cytoplasmic	u	d			+	[1]
15	ACLY	ATP-citrate synthase	u	d			+	[2]
9	ACTA2	Actin, aortic smooth muscle	u	d			+	[3]
6	ACTB	Actin, cytoplasmic 1	u	d			+	[4]
7	ACTBL2	Beta-actin-like protein 2	u	d			+	[4]
8	ACTN1	Alpha-actinin-1 (f-actin cross linking protein)	u	d			+	[5]
7	ACTN4	Alpha-actinin-4	u	d			+	[5]
2	ACTR2	Actin-related protein 2	u	d			+	[6]
2	ACTR3	Actin-related protein 3	u				+	[7]
3	AFP	Alpha-fetoprotein		d			+	[8]
15	AHCY	Adenosylhomocysteinase		d			+	
4	AHSA1	Activator of 90 kDa heat shock protein ATPase homolog 1		d			+	
5	ALDH18A1	Delta-1-pyrroline-5-carboxylate synthetase		d			+	
3	ALDOA	Fructose-bisphosphate aldolase A	u	d			+	[9]
4	ALDOC	Fructose-bisphosphate aldolase C	u	d			+	[10]
2	ANP32A	Acidic leucine-rich nuclear phosphoprotein 32 family member A	u	d			+	
4	ANP32B	Acidic leucine-rich nuclear phosphoprotein 32 family member B		d	N		+	[11]
2	ANP32C	Acidic leucine-rich nuclear phosphoprotein 32 family member C					+	
8	ANXA5	Annexin A5	u	d			+	[12]
5	ANXA6	Annexin A6	u	d			+	[13]
4	APEH	Acylamino-acid-releasing enzyme					+	
2	APEX1	DNA-(apurinic or apyrimidinic site) lyase	u	d			+	[14]
2	API5	Apoptosis inhibitor 5		d			+	
8	ARF1	ADP-ribosylation factor 1			Nsp6		+	
6	ARHGDIA	Rho GDP-dissociation inhibitor 1	u	d			+	
8	ARHGDIB	Rho GDP-dissociation inhibitor 2		d			+	[15]
3	ARPC2	Actin-related protein 2/3 complex subunit 2		d			+	
2	ASNS	Asparagine synthetase [glutamine-hydrolyzing]	u				+	
14	ATIC	Bifunctional purine biosynthesis protein, PURH					+	[16]
13	ATP5F1B	ATP synthase subunit beta, mitochondrial, ATP5B	u	d	Nsp6	+		[17]
3	ATXN10	Ataxin-10, Spinocerebellar ataxia type 10 protein	u	d			+	
2	BZW1	Basic leucine zipper and W2 domain-containing protein 1	u				+	
3	BZW2	Basic leucine zipper and W2 domain-containing protein 2			N		+	
2	C1QBP	Complement component 1 Q subcomponent-binding protein		d			+	[18]
8	CALM1	Calmodulin	u	d			+	[19]
13	CALR	Calreticulin	u	d			+	[20]
15	CAND1	Cullin-associated NEDD8-dissociated protein 1					+	
6	CAP1	Adenylyl cyclase-associated protein 1	u	d			+	
3	CAPN1	Calpain-1 catalytic subunit					+	
2	CAPRIN1	Caprin-1, Cell cycle associated protein 1		d			+	
3	CAPZA1	F-actin capping protein subunit alpha-1 (capz alpha-1)		d			+	[21]
3	CAPZB	F-actin-capping protein subunit beta		d			+	[22]
10	CCT2	T-complex protein 1 subunit beta (tcp-1-beta) (cct-beta)		d	Nsp12 Orf8 Orf9b Orf10		+	[23]
6	CCT3	T-complex protein 1 subunit gamma (chaperonin containing TCP1, subunit 3 isoform)	u		Orf8 Orf10		+	[24]
6	CCT4	T-complex protein 1 subunit delta (tcp-1-delta) (cct-delta) (stimulator of tar rna-binding)	u				+	[24]
10	CCT5	T-complex protein 1 subunit epsilon (tcp-1-epsilon) (cct-epsilon)	u	d	Nsp1 Nsp12 Orf8 Orf10		+	[23]
7	CCT6A	T-complex protein 1 subunit zeta (acute morphine dependence-related protein 2)	u	d	Nsp1 Nsp12 Orf10		+	[23]
5	CCT7	T-complex protein 1 subunit eta (hiv-1 nef-interacting protein)			Orf10		+	[23]
20	CCT8	T-complex protein 1 subunit theta	u	d	Nsp1 Nsp12 Nsp14		+	[24]
4	CDC37	Hsp90 co-chaperone, hsp90 chaperone protein kinase-targeting	u	d	Nsp16		+	
6	CLIC1	Chloride intracellular channel protein 1	u	d	Nsp16		+	[25]
12	CLTC	Clathrin heavy chain 1	u	d			+	[26]
4	CLTCL1	Clathrin heavy chain 2					+	
4	CLUH	Clustered mitochondria protein homolog (mRNA-binding)		d	Nsp7 Nsp16		+	
2	CMPK1	UMP-CMP kinase		d			+	
3	CNDP2	Cytosolic non-specific dipeptidase	u		Orf3 Orf10		+	
2	CNPY2	Protein canopy homolog 2, MSAP, TMEM4, Zsig9		d	Orf3a		+	
2	C0PZ1	Coatomer subunit zeta-1		d			+	
12	C0R01A	Coronin-1A	u				+	[27]
3	CPNE1	Copine-1					+	
4	CPNE3	Copine-3	u	d			+	
3	CS	Citrate synthase, mitochondrial	u	d			+	[2]
10	CSE1L	Exportin-2		d			+	
4	CSK	Tyrosine-protein kinase CSK		d			+	[28]
6	DARS	Aspartate-tRNA ligase, cytoplasmic, DARS1					+	[29]
3	DCK	Deoxycytidine kinase	u				+	
3	DCTN2	Dynactin subunit 2			Orf6		+	
3	DCTPP1	dCTP pyrophosphatase 1		d			+	
7	DDB1	DNA damage-binding protein 1	u	d			+	[26]
4	DDX39A	ATP-dependent RNA helicase DDX39A	u	d			+	
3	DDX39B	Spliceosome RNA helicase DDX39B		d			+	
2	DDX5	Probable ATP-dependent RNA helicase, RNA helicase p68	u	d			+	[30]
2	DHX15	Pre-mRNA-splicing factor ATP-dependent RNA helicase DHX15		d			+	
3	DHX9	ATP-dependent RNA helicase A					+	[31]
3	DIABL0	Diablo homolog, mitochondrial	u	d	Nsp6 Nsp15		+	
4	DLD	Dihydrolipoyl dehydrogenase, mitochondrial					+	[32]
2	DNAJC8	DnaJ homolog subfamily C member 8	u				+	
4	DNPH1	2’-deoxynucleoside 5’-phosphate N-hydrolase 1	u				+	
6	DPP3	Dipeptidyl-peptidase 3		d			+	
5	DUT	Deoxyuridine 5’-triphosphate nucleotidohydrolase, mitochondrial	u	d			+	
2	EEF1A1	Elongation factor 1-alpha	u	d			+	[33]
4	EEF1A2	Elongation factor 1-alpha 2	u		Orf3		+	[34]
2	EEF1B2	Elongation factor 1-beta		d			+	
5	EEF1D	Elongation factor 1-delta		d			+	[35]
6	EEF1G	Elongation factor 1-gamma	u	d			+	
17	EEF2	Elongation factor 2	u	d			+	[36]
2	EFTUD2	U5 small nuclear ribonucleoprotein component 116 kda		d			+	[37]
3	EIF2A	Eukaryotic translation initiation factor 2 subunit alpha, EIF2S1					+	
5	EIF3A	Eukaryotic translation initiation factor 3 subunit A	u	d	Nsp1		+	[38]
5	EIF3B	Eukaryotic translation initiation factor 3 subunit B	u	d	Nsp1		+	
2	EIF3C	Eukaryotic translation initiation factor 3 subunit C		d	Nsp1			[39]
3	EIF3E	Eukaryotic translation initiation factor 3 subunit E (viral integration site protein int-6 homolog)	u	d	Nsp1		+	[40]
4	EIF3F	Eukaryotic translation initiation factor 3 subunit F	u	d	Nsp1		+	
2	EIF3I	Eukaryotic translation initiation factor 3 subunit I		d	Nsp1		+	[39]
10	EIF3L	Eukaryotic translation initiation factor 3 subunit L (subunit E interacting protein)		d	Nsp1		+	
19	EIF4A1	Eukaryotic initiation factor 4A-I	u	d			+	
4	EIF4A3	Eukaryotic initiation factor 4A-III					+	[41]
2	EIF5	Eukaryotic translation initiation factor 5	u	d			+	[42]
5	EIF5A	Eukaryotic translation initiation factor 5A-1	u	d			+	[42]
2	EIF6	Eukaryotic translation initiation factor 6	u				+	
3	ELAVL1	ELAV-like protein 1		d			+	[43]
3	EN02	Gamma-enolase	u	d			+	[44]
2	EPRS	Bifunctional glutamate/proline-tRNA ligase	u				+	[45]
4	ER01L	ER01-like protein alpha		d	Orf3a		+	
4	ETF1	Eukaryotic peptide chain release factor subunit 1	u				+	
14	EZR	Ezrin	u	d	S		+	[46]
3	FARSB	Phenylalanine-tRNA ligase beta subunit					+	[47]
19	FASN	Fatty acid synthase	u	d			+	[48]
2	FDPS	Farnesyl pyrophosphate synthetase like-4 protein		d			+	
2	FEN1	Flap endonuclease 1	u	d			+	
2	FERMT3	Fermitin family homolog 3	u				+	
11	FKBP4	Peptidyl-prolyl cis-trans isomerase FKBP4, FKBP-52			Nsp12		+	[49]
2	FKBP5	Peptidyl-prolyl cis-trans isomerase FKBP5 (FK506-binding protein)	u				+	
2	FUBP1	Far upstream element-binding protein 1	u	d			+	
2	G6PD	Glucose-6-phosphate 1-dehydrogenase	u	d			+	[50]
5	GANAB	Neutral alpha-glucosidase AB		d	Orf6 Orf8 Orf9c		+	[51]
6	GAPDH	Glyceraldehyde-3-phosphate dehydrogenase	u	d	Orf8		+	[52]
4	GARS	Glycine-tRNA ligase, GARS1	u				+	[53]
2	GART	Trifunctional purine biosynthetic protein adenosine-3		d	Nsp15		+	
2	GBE1	1,4-alpha-glucan-branching enzyme	u				+	
8	GDI1	Rab GDP dissociation inhibitor alpha	u	d			+	[54]
10	GDI2	Rab GDP dissociation inhibitor beta	u	d	Nsp12 Orf9b		+	[55]
2	GGCT	Gamma-glutamylcyclotransferase, cytochrome c-releasing factor 21	u				+	
3	GL01	Lactoylglutathione lyase		d	Orf3		+	[56]
3	GLRX3	Glutaredoxin-3		d			+	
10	GLUD1	Glutamate dehydrogenase 1, mitochondrial					+	[57]
2	GMFB	Glia maturation factor, beta	u				+	
3	GSPT1	Eukaryotic peptide chain release factor GTP-binding subunit ERF3A					+	
3	GSS	Glutathione synthetase		d			+	
2	GTF2I	General transcription factor II-I	u	d			+	
3	H2AFV	Histone H2A.V, H2AZ2	u	d		+		[58]
2	H2AFY2	Core histone marco-H2A.1, MARC0H2A1				+		[59]
3	HARS	Histidine-tRNA ligase, cytoplasmic					+	[19]
3	HDGF	Hepatoma-derived growth factor, hmg-1l2	u	d			+	[60]
5	HIST1H1C	Histone 1.2, H1–2	u	d	Nsp8		+	[61]
2	HIST1H2AB	Histone H2A type 1-B/E, H2AC4		d		+		[62]
2	HIST1H2BL	Histone H2B type 1-L, H2BC13	u			+		[63]
14	HIST1H4J	Histone H4, H4C1				+		[64]
9	HIST2H2BE	Histone H2B type 2-E, H2BC21	u	d		+		[65]
5	HIST2H3A	Histone H3.2, H3C15	u	d		+		[66]
4	HMGB1	High mobility group protein 1-like 10 (hmg-1l10)		d			+	[60]
3	HMGCS1	Hydroxymethylglutaryl-CoA synthase, cytoplasmic	u	d			+	
4	HNRNPA1	Heterogeneous nuclear ribonucleoprotein A1	u	d			+	[67]
8	HNRNPA2B1	Heterogeneous nuclear ribonucleoproteins A2/B1	u	d			+	[68]
2	HNRNPA3	Heterogeneous nuclear ribonucleoprotein A3	u	d			+	[69]
2	HNRNPAB	Heterogeneous nuclear ribonucleoprotein A/B		d			+	[69]
3	HNRNPC	Heterogeneous nuclear ribonucleoprotein C1/C2	u	d			+	[70]
5	HNRNPCL1	Heterogeneous nuclear ribonucleoprotein C-like 1					+	[70]
5	HNRNPD	Heterogeneous nuclear ribonucleoprotein D					+	[71]
2	HNRNPDL	Heterogeneous nuclear ribonucleoprotein D-like	u	d			+	[72]
3	HNRNPF	Heterogeneous nuclear ribonucleoprotein F		d			+	[73]
2	HNRNPH1	Heterogeneous nuclear ribonucleoprotein H (hnrnp h)	u	d			+	[74]
9	HNRNPK	Heterogeneous nuclear ribonucleoprotein K (transformation up-regulated nuclear protein)	u				+	[75]
5	HNRNPR	Heterogeneous nuclear ribonucleoprotein R	u	d			+	[76]
2	HNRNPU	Heterogeneous nuclear ribonucleoprotein U	u	d			+	[77]
6	HPRT1	Hypoxanthine-guanine phosphoribosyltransferase					+	
16	HSP90AA1	Heat shock protein HSP 90-alpha (hsp 86) (ny-ren-38 antigen)	u	d				[78]
6	HSP90AA2	Heat shock protein HSP 90-alpha A2	u				+	[79]
16	HSP90AB1	Heat shock protein HSP 90-beta (hsp 84) (hsp 90)	u	d	Nsp12		+	[80]
19	HSP90B1	Endoplasmin, GRP94, tumor rejection antigen 1	u	d	Orf3a Orf9c		+	[79]
4	HSPA4	Heat shock 70 kda protein 4	u	d			+	[81]
15	HSPA5	Endoplasmic reticulum chaperone, GRP78, BiP	u	d	S E M Nsp2 Nsp4 Nsp6 Orf3a Orf7a Orf7b		+	[82]
24	HSPA8	Heat shock cognate 71 kDa protein	u	d	Nsp2 Nsp12		+	[83]
12	HSPA9	Stress-70 protein, mitochondrial (grp 75)	u	d	N		+	[84]
19	HSPD1	60 kda heat shock protein, mitochondrial matrix protein p1	u	d			+	[85]
4	HSPH1	Heat shock protein 105 kD	u				+	[86]
2	IDE	Insulin-degrading enzyme			Nsp4 Nsp12 Nsp14 Nsp15 Nsp16 Orf3b		+	
2	IDH3A	Isocitrate dehydrogenase [NAD] subunit alpha, mitochondrial					+	
6	ILF2	Interleukin enhancer-binding factor 2	u				+	[87]
6	IP05	Importin-5, KPNB3, RANBP5					+	[88]
3	IP07	Importin-7, RANBP7			Nsp6 Orf9c		+	[89]
8	IQGAP1	Ras GTPase-activating-like protein IQGAP1	u				+	[90]
4	KARS	Lysine-tRNA ligase, KARS1			Nsp7		+	[45]
3	KHSRP	Far upsteam element-binding protein 2 (KH-type splicing regulatory protein)	u	d			+	
2	KPNA2	Importin subunit alpha-1		d	Orf6		+	
11	KPNB1	Importin subunit beta-1					+	[88]
30	LCP1	Plastin-2 (l-plastin) (lymphocyte cytosolic protein 1)	u	d			+	[91]
7	LDHA	L-lactate dehydrogenase A chain (proliferation-inducing gene 19 protein)	u	d	Nsp12		+	[92]
10	LDHB	L-lactate dehydrogenase B chain	u	d	Nsp12 Nsp7		+	[93]
16	LRPPRC	leucine-rich PPR motif-containing protein		d			+	[94]
7	MCM2	DNA replication licensing factor MCM2		d			+	[95]
3	MCM3	DNA replication licensing factor mcm3 (dna polymerase alpha holoenzyme-associated protein p1)(p102 protein) (p1-mcm3)	u	d			+	[95]
3	MCM4	DNA replication licensing factor mcm4 (cdc21 homolog) (p1-cdc21)	u	d			+	[95]
2	MCM6	DNA replication licensing factor mcm6 (p105mcm)	u	d			+	[95]
2	ME2	NAD-dependent malic enzyme, mitochondrial	u	d	Nsp15		+	
6	MSN	Moesin	u		S Nsp6 Orf3		+	[96]
2	MYG1	UPF0160 protein MYG1, mitochondrial, C12orf10					+	
4	MZB1	Marginal zone B- and B1-cell-specifc protein (Proapoptotic caspase adapter protein, plasma cell-induced resident protein)					+	
3	NAA15	N-alpha-acetyltransferase 15, NatA auxiliary subunit (NMDA receptor-regulated protein, NARG1)		d			+	
2	NAA25	N-alph-acetyltransferase 25, NatB auxiliary subunit (TPR repeat-containing protein C12orf30)					+	
3	NACA	Nascent polypeptide-associated complex subunit alpha (nac-alpha)	u	d			+	[97]
7	NAP1L1	Nucleosome assembly protein 1-like 1 (nap-1-related protein)(hnrp)		d			+	
4	NAP1L4	Nucleosome assembly protein 1-like 4 (nucleosome assembly protein 2)	u	d			+	
5	NARS	Asparagine-tRNA ligase, cytoplasmic, NARS1		d			+	[98]
4	NASP	Nuclear autoantigenic sperm protein (histone binding protein)	u	d			+	[99]
12	NCL	Nucleolin	u	d			+	[100]
8	NME1	Nucleoside diphosphate kinase A		d			+	[101]
15	NPEPPS	Puromycin-sensitive aminopeptidase					+	
4	NPM1	Nucleophosmin	u	d	Orf9c		+	[102]
3	NSFL1C	NSFL1 cofactor p47	u				+	
8	NUDC	Nuclear migration protein nudC (nuclear distribution protein c homolog)		d	Nsp12		+	
2	NUDT21	Cleavage and polyadenylation specificity factor subunit 5		d			+	
2	NUDT3	Diphosphoinositol polyphosphate phosphohydrolase					+	
4	NUDT5	ADP-sugar pyrophosphatase (nucleoside diphosphate-linked moiety x motif 5) (nudix motif 5)		d			+	
2	0TUB1	Ubiquitin thioesterase protein 0TUB1					+	
5	P4HB	Protein disulfide-isomerase (cellular thyroid hormone-binding protein) (p55)	u	d	Nsp7 Orf3b		+	[103]
14	PA2G4	Proliferation-associated protein 2G4	u				+	
9	PABPC1	Polyadenylate-binding protein 1		d	N	+		[104]
2	PAFAH1B2	Platelet-activating factor acetylhydrolase IB subunit beta	u	d			+	
2	PAFAH1B3	Platelet-activating factor acetylhydrolase IB subunit gamma	u		Nsp12 Nsp5 Orf3b		+	
6	PAICS	Multifunctional protein ADE2		d			+	
2	PARP1	Poly [ADP-ribose] polymerase 1	u	d			+	
8	PCNA	Proliferating cell nuclear antigen (cyclin)	u	d			+	[52]
2	PDCD10	Programmed cell death protein 10					+	
21	PDIA3	Protein disulfide-isomerase A3	u	d	M Orf3a Orf3b Orf8 Orf9c		+	[105]
8	PDIA4	Protein disulfide-isomerase A4 (protein erp-72) (erp72)	u	d	Nsp16 Nsp7 Orf3b		+	[106]
5	PDIA6	Protein disulfide-isomerase A6 (protein disulfide isomerase p5) (thioredoxin domain-containing protein 7)	u	d			+	[103]
7	PFAS	Phosphoribosylformylglycinamidine synthase			Nsp12 Nsp15 Nsp16 Nsp7		+	
3	PFDN2	Prefoldin subunit 2	u		Nsp12 Nsp15 Orf10		+	[107]
2	PFDN3	Prefoldin subunit 3, von hippel-lindau-binding protein 1, VBP1		d	Nsp12 Nsp15		+	
9	PFN1	Profilin-1 (profilin i)	u	d			+	[95]
4	PGAM1	Phosphoglycerate mutase 1	u	d			+	[108]
4	PGAM2	Phosphoglycerate mutase 2					+	
3	PGLS	6-phosphogluconolactonase	u				+	
3	PHGDH	D-3-phosphoglycerate dehydrogenase	u	d			+	[109]
10	PLCG2	1-phosphatidylinositol-4,5-bisphosphate phosphodiesterase gamma-2	u				+	
2	PMPCB	Mitochondrial-processing peptidase subunit beta		d	M		+	
8	PPA1	Inorganic pyrophosphatase	u		Orf3		+	[110]
3	PPAT	Amidophosphoribosyltransferase		d			+	
6	PPIB	Peptidyl-prolyl cis-trans isomerase B	u	d	Orf8		+	[111]
2	PPP1R7	Protein phosphatase 1 regulatory subunit 7 (protein phosphatase 1 regulatory subunit 7) (protein phosphatase 1 regulatory subunit 22)	u				+	
3	PPP2R1A	Serine/threonine-protein phosphatase 2A 65 kDa regulatory subunit A alpha isoform		d			+	
3	PRDX1	Peroxiredoxin-1	u	d			+	[112]
5	PRDX3	Thioredoxin-dependent peroxide reductase, mitochondrial	u	d			+	[113]
2	PRKCSH	Glucosidase 2 subunit beta (protein kinase c substrate)		d	S Nsp6 Orf3 Orf3a		+	
2	PRMT1	Protein arginine n-methyltransferase 1 (hnRNP methyltransferase-like 2 isoform (ec 2.1.1.-) (interferon receptor 1-bound protein 4)		d			+	[114]
2	PRPSAP2	Phosphoribosyl pyrophosphate synthetase-associated protein 2	u				+	
3	PSMA1	Proteasome subunit alpha type-1		d	Orf3b		+	[115]
2	PSMA2	Proteasome subunit alpha type-2		d			+	
6	PSMA3	Proteasome subunit alpha type-3 (macropain subunit c8)	u	d	Nsp2 Nsp4 Nsp7 Nsp10 Nsp12		+	[116]
5	PSMA4	Proteasome subunit alpha type-4 (C9)	u				+	[117]
5	PSMA5	Proteasome subunit alpha type-5 (macropain zeta chain)	u		Orf3b		+	[118]
8	PSMA6	Proteasome subunit alpha type-6	u	d	Orf3b		+	
4	PSMA7	Proteasome subunit alpha type-7	u	d			+	[119]
5	PSMB1	Proteasome subunit beta type-1					+	[120]
3	PSMB3	Proteasome subunit beta type-3 (proteasome theta chain)		d	Orf3b		+	[116]
7	PSMB4	Proteasome subunit beta type-4 (proteasome beta chain) (macropain beta chain) (multicatalytic endopeptidase complex beta chain)			Orf3b		+	
2	PSMB6	Proteasome subunit beta type-6 (proteasome delta chain)(multicatalytic endopeptidase complex delta chain)		d	Orf3b		+	
3	PSMB7	Proteasome subunit beta type-7 (proteasome subunit z)		d			+	[121]
3	PSMB8	Proteasome subunit beta type-8	u	d			+	
4	PSMB9	Proteasome subunit beta type-9	u	d			+	
2	PSMC3	26S protease regulatory subunit 6A		d	Orf6		+	
5	PSMD1	26S proteasome non-ATPase regulatory subunit 1	u	d	Nsp7 Orf6 Orf8		+	
9	PSMD11	Proteasome 26S non-ATPase regulatory subunit 11	u				+	
3	PSMD12	26S proteasome non-ATPase regulatory subunit 12		d			+	
2	PSMD14	26S proteasome non-ATPase regulatory subunit 14					+	
8	PSMD3	26S proteasome non-ATPase regulatory subunit 3		d			+	
3	PSMD6	26S proteasome non-ATPase regulatory subunit 6					+	
11	PSME1	Proteasome activator complex subunit 1	u		Nsp15		+	
8	PSME2	Proteasome activator complex subunit 2	u				+	
4	PSME3	Proteasome activator complex subunit 3		d	Nsp16		+	[122]
2	PSPH	Phosphoserine phosphatase					+	
3	PTBP1	Polypyrimidine tract-binding protein 1	u	d			+	[123]
2	PTBP3	Polypyrimidine tract-binding protein (cDNA FLJ51619, highly similar to Regulator of differentiation) ROD1	u	d			+	
2	PTGES3	Prostaglandin E synthase 3 (telomerase-binding protein p23) (hsp90 co-chaperone) (progesterone rec)		d			+	
2	PTPN6	Tyrosine-protein phosphatase non-receptor type 6	u	d			+	
2	PUS1	tRNA pseudouridine synthase A					+	
5	RAB7A	Ras-related protein Rab-7a	u	d	Nsp7 Orf3 Orf7b		+	
2	RAD23A	UV excision repair protein RAD23 homolog A		d			+	[124]
3	RANBP1	RanBP	u	d			+	
2	RANBP6	Ran-binding protein 6		d	Orf7a		+	
7	RDX	Radixin, isoform CRA (actin binding to plasma membrane)	u	d	Nsp13		+	[125]
2	RPL10A	60S ribosomal protein L10a				+		
2	RPL11	60S ribosomal protein L11	u				+	
2	RPL15	60S ribosomal protein L15		d		+		
2	RPL22	60s ribosomal protein L22 (Epstein-Barr virus small rna-associated protein)(heparin-binding protein hbp15)		d			+	[121]
4	RPLP0	60s acidic ribosomal protein P0	u	d		+		[126]
2	RPS15A	40s ribosomal protein S15a	u					
2	RPS25	40S ribosomal protein S25	u	d	Orf8		+	
3	RPS27A	Ubiquitin-40S ribosomal protein S27a	u	d	S Nsp4	+		[121]
6	RPS3	40S ribosomal protein S3	u	d	Orf8		+	[127]
3	RPS3A	40S ribosomal protein S3a	u	d	Orf8		+	
3	RPS7	40S ribosomal protein S7	u	d			+	
5	RPSA	similar to 40S ribosomal protein SA (p40) (34/67 kDa laminin receptor) (Colon carcinoma laminin-binding protein) (NEM/1CHD4) (Multidrug resistance-associated protein MGr1-Ag), RPSAP12					+	
6	RUVBL1	RuvB-like 1, tata box-binding protein-interacting protein, nuclear matrix protein 238			Nsp1 Nsp7 Nsp12 Orf9b		+	[128]
5	RUVBL2	RuvB-like 2		d	Nsp1 Nsp7 Nsp12		+	[129]
2	SARS	Serine-tRNA ligase, cytoplasmic, SARS1			Nsp15		+	
2	SEPT11	Septin-11		d			+	
2	SEPT2	Septin-2, NEDD5, DIFF6					+	[130]
3	SEPT7	Septin-7		d			+	[131]
8	SERPINB9	Serpin B9	u	d			+	
2	SERPINC1	Antithrombin-III	u				+	
6	SET	Protein SET (phosphatase 2a inhibitor i2pp2a) (i-2pp2a) (template-activating factor i) (taf-i) (hla-dr-associated protein ii) (phapii)	u	d			+	[132]
3	SF3B3	Splicing factor 3B subunit 3					+	[133]
2	SFPQ	Splicing factor, proline- and glutamine-rich	u	d			+	[134]
3	SGTA	Small glutamine-rich tetratricopeptide repeat-containing protein alpha	u	d	M		+	
2	SHMT1	Serine hydroxymethyltransferase, cytosolic		d			+	
9	SHMT2	Serine hydroxymethyltransferase, mitochondrial		d			+	
2	SLIRP	SRA stem-loop-interacting RNA-binding protein, mitochondrial	u	d			+	
8	SND1	Staphylococcal nuclease domain-containing protein 1	u	d			+	
2	SNRNP70	U1 small nuclear ribonucleoprotein 70 kDa	u	d			+	[135]
3	SNRPA	U1 small nuclear ribonucleoprotein A	u				+	[136]
3	SNRPA1	U2 small nuclear ribonucleoprotein A′					+	[137]
2	SNRPD1	Small nuclear ribonucleoprotein Sm D1	u				+	[138]
2	SNRPD2	Small nuclear ribonucleoprotein Sm D2		d			+	[139]
2	SNRPD3	Small nuclear ribonucleoprotein Sm d3 (snrnp core protein d3) (sm-d3)		d		+		[138]
2	SNRPE	Small nuclear ribonucleoprotein E		d			+	[140]
2	SPTAN1	Spectrin alpha chain, non-erythrocytic 1	u	d			+	[141]
3	SRM	Spermidine synthase		d			+	
3	SRP14	Signal recognition particle 14 kDa protein	u	d	Nsp13 Orf8		+	
3	SRSF1	Serine/argine-rich splicing factor 1 (Isoform ASF-1 of Splicing factor, arginine/serine-rich	u	d			+	[142]
3	SRSF2	Arginine/serine-rich splicing factor 2, SFRS2	u	d			+	[143]
3	SSB	Lupus La protein (Sjoegren syndrome type b antigen) (La/SSB)	u	d			+	[144]
4	SSBP1	Single-stranded DNA-binding protein, mitochondrial			N	+		
4	ST13	Hsc70-interacting protein (hip) (suppression of tumorigenicity protein 13) (putative tumor suppressor st13) (protein fam10a1) (progesterone receptor-associate)	u		Nsp12 Orf3b Orf6 Orf8 Orf10		+	[145]
3	STIP1	Stress-induced-phosphoprotein 1	u	d	Nsp12 Orf3a Orf8 E		+	
2	SUGT1	Protein SGT1 homolog (Suppressor of G2 allele of SKP1 homolog)	u		Nsp12 Nsp15		+	
2	SWAP70	Switch-associated protein 70		d	Nsp2		+	
5	SYNCRIP	Heterogeneous nuclear ribonucleoprotein Q		d			+	
2	TALDO1	Transaldolase	u	d			+	[146]
3	TBCA	Tubulin-specific chaperone A			Nsp11		+	
3	TCL1A	T-cell leukemia/lymphoma protein 1A	u	d			+	
7	TCP1	T-complex protein 1 subunit alpha (tcp-1-alpha) (cct-alpha)		d	Orf10		+	[23]
10	TLN1	Talin-1	u	d			+	[147]
5	TNPO1	Transportin-1					+	
2	TOP1	DNA topoisomerase 1 (Scl 70)	u			+		[148]
5	TPM1	Tropomyosin alpha-1 chain isoform	u	d	Nsp9		+	[149]
6	TPM3	Tropomyosin alpha-3 chain (tropomyosin gamma)	u	d			+	[150]
5	TPM4	Tropomyosin alpha-4 chain	u	d			+	[151]
4	TPP2	Tripeptidyl-peptidase 2					+	
3	TTLL12	Tubulin-tyrosine ligase-like protein 12		d			+	[152]
2	TTLL3	Tubulin monoglycylase TTLL3	u				+	
4	TUBA1C	TUBA1C protein	u	d		+		[153]
12	TUBA4A	Tubulin alpha-4A chain	u	d			+	[154]
5	TUBB	Tubulin beta chain	u	d			+	[155]
4	TUBB1	Tubulin beta-1 chain					+	[156]
2	TUBB4	Tubulin beta-3 chain, beta-4 chain	u	d			+	[157]
12	TUBB4B	Tubulin beta-4B chain (Tubulin beta-2C chain)	u	d			+	[156]
2	TXNDC17	Thioredoxin domain-containing protein 17	u	d			+	
4	TXNRD1	Thioredoxin reductase 1, cytoplasmic	u	d			+	[158]
2	U2AF2	Splicing factor U2AF 65 kDa subunit		d			+	
7	UBA1	Ubiquitin-like modifier-activating enzyme 1 (Ubiquitin-activating enzyme E1)	u	d			+	[159]
2	UBA6	Ubiquitin-like modifier-activating enzyme 6			Nsp7		+	
2	UBE2K	Ubiquitin-conjugating enzyme E2 K					+	
3	USP5	Ubiquitin carboxyl-terminal hydrolase 5	u	d			+	
3	VARS1	Valine-tRNA ligase					+	
12	VCP	Transitional endoplasmic reticulum ATPase (valosin-containing protein)	u	d			+	[160]
10	VIM	Vimentin	u	d		+		[161]
6	WARS	Tryptophan-tRNA ligase, cytoplasmic, WARS1	u	d			+	[162]
2	XPNPEP1	Xaa-Pro aminopeptidase 1		d			+	
4	XPO1	Exportin-1			Nsp4 Orf7a		+	
5	XPOT	Exportin-T (trna exportin) (exportin(trna))	u		Orf7a		+	
3	XRCC5	X5-ray repair cross-complementing protein 5, lupus ku86 ku80 autoantigen		d			+	[163]
9	XRCC6	X-ray repair cross-complementing protein 6 (ku70)	u	d			+	[164]
6	YARS	Tyrosine-tRNA ligase, cytoplasmic, YARS1	u	d			+	[165]
5	YWHAB	14-3-3 protein beta/alpha (protein kinase c inhibitor protein 1)	u	d			+	
12	YWHAE	14-3-3 protein epsilon	u	d			+	[166]
3	YWHAG	14-3-3 protein gamma	u	d			+	[166]
3	YWHAH	14-3-3 protein eta (protein as1)		d			+	[167]
3	YWHAQ	14-3-3 protein theta	u	d			+	[168]
7	YWHAZ	14-3-3 protein zeta/delta	u	d			+	[169]
2	ZPR1	Zinc finger protein ZPR1	u	d			+	[170]
